# Supplementary material for: Comparative multiomics analysis of cell physiological state after culture in a basket bioreactor
Source: Sci Rep. 2022 Nov 23;12:20161. doi: 10.1038/s41598-022-24687-4 (PMC9686226; doi:10.1038/s41598-022-24687-4)

66048.1:132761196:132761385:-@NW_023666048.1:132761726:132763223:-@NW_023666048.1:132763124:132

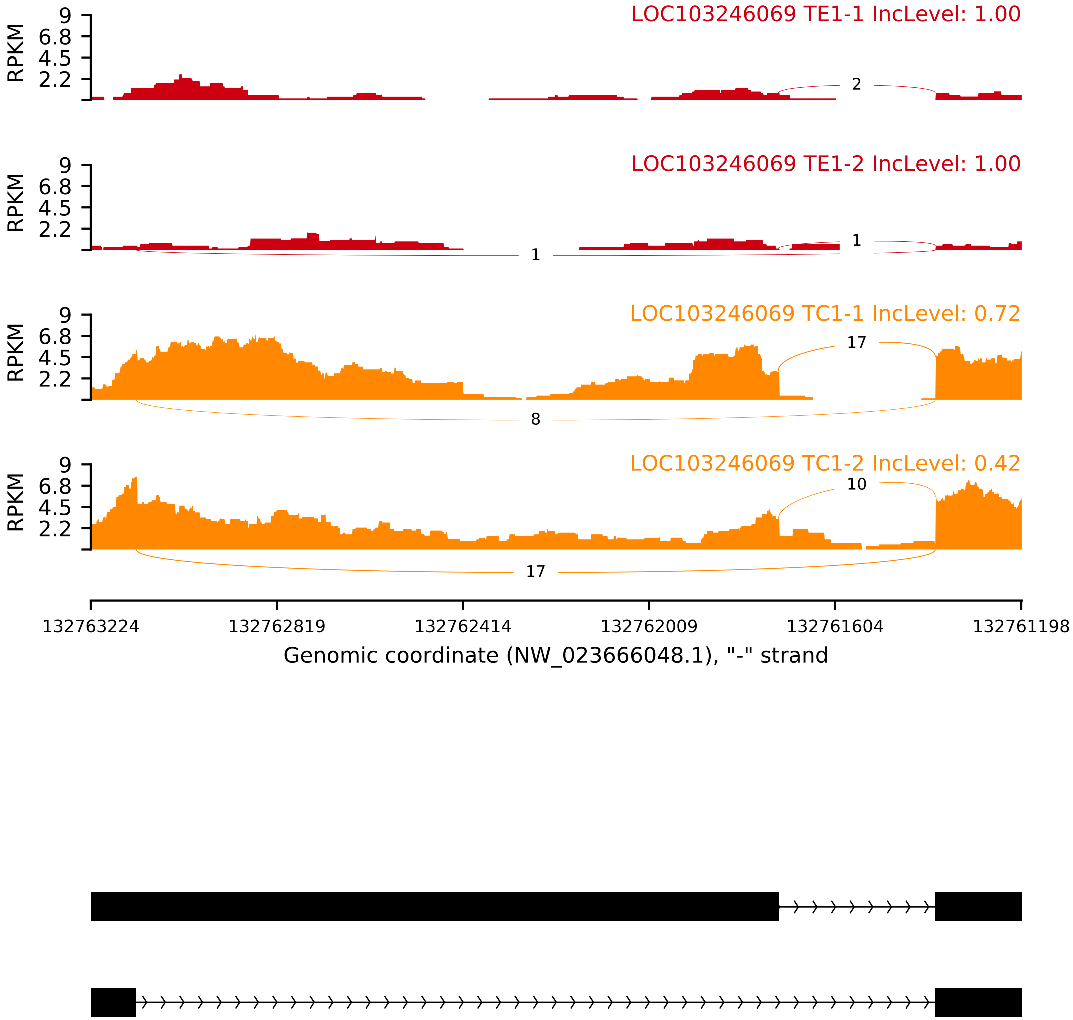

Supplement: Supplementary file 1 — Supplementary Information 1. [file 41598_2022_24687_MOESM1_ESM.zip › raw data/Transcriptomic raw data/7.AS/2.dasplot/TE1vsTC1/A5SS/14_LOC103246069_NW_023666048.1_132761196_132761385_-@NW_023666048.1_132761726_132763223_-@NW_023666048.1_132763124_132763223_-.pdf]
